# Metyrapone Versus Osilodrostat in the Short-Term Therapy of Endogenous Cushing’s Syndrome: Results From a Single Center Cohort Study

**DOI:** 10.3389/fendo.2022.903545

**Published:** 2022-06-13

**Authors:** Mario Detomas, Barbara Altieri, Timo Deutschbein, Martin Fassnacht, Ulrich Dischinger

**Affiliations:** ^1^ Department of Internal Medicine I, Division of Endocrinology and Diabetes, University Hospital Würzburg, University of Würzburg, Würzburg, Germany; ^2^ Medicover Oldenburg MVZ, Oldenburg, Germany; ^3^ Comprehensive Cancer Center Mainfranken, University of Würzburg, Würzburg, Germany

**Keywords:** metyrapone, osilodrostat, Cushing’s syndrome, hypercortisolism, medical therapy, blood pressure, isturisa, efficacy

## Abstract

**Background:**

Although surgery is considered the first-line treatment for patients with endogenous Cushing’s syndrome (CS), medical therapy is often required to control severe hypercortisolism. Metyrapone and osilodrostat are both steroidogenic inhibitors targeting the 11β-hydroxylase, however, their therapeutic effectiveness has not yet been directly compared. This study aimed to evaluate metyrapone and osilodrostat in the short-term therapy of CS.

**Methods:**

Retrospective analysis of patients with endogenous CS treated with metyrapone or osilodrostat as monotherapy for at least 4 weeks. Main outcome measures were serum cortisol and 24h urinary free cortisol (UFC) at baseline (T0) and after 2 (T1), 4 (T2), and 12 weeks (T3) of therapy.

**Results:**

16 patients with endogenous CS were identified (pituitary n=7, adrenal n=4, ectopic CS n=5). Each 8 patients were treated with metyrapone and osilodrostat. Despite heterogeneity, both groups showed comparable mean UFC levels at T0 (metyrapone: 758 µg/24h *vs* osilodrostat: 817 µg/24h; *p*=0.93). From T0 to T1, the decrease of UFC was less pronounced under metyrapone than osilodrostat (-21.3% *vs* -68.4%; median daily drug dose: 1000 mg vs 4 mg). This tendency persisted at T2 (-37.3% vs -50.1%; median drug dose: 1250 mg *vs* 6 mg) while at T3 a decrease in UFC from T0 was more pronounced in the metyrapone group (-71.5% *vs* -51.5%; median dose 1250 mg *vs* 7 mg). Under osilodrostat, a QTc-interval prolongation was identified at T3 (mean 432 ms vs 455 ms). From T0 to T2, the number of antihypertensive drugs remained comparable under metyrapone and decreased under osilodrostat (n= -0.3 *vs* n= -1.0).

**Conclusion:**

Although both drugs show comparable therapeutic efficacy, osilodrostat seems to reduce cortisol levels and to control blood pressure faster.

## 1 Introduction

Endogenous Cushing’s syndrome (CS) is a rare disorder with an incidence of 0.2–5.0 per million people per year (1). If the underlying glucocorticoid excess is not properly diagnosed and rapidly treated, it may lead to several comorbidities and increased mortality (2–4).

Surgery is considered the first-line treatment for patients with endogenous CS (2), e.g. transsphenoidal adenomectomy in Cushing’s disease (CD) or adrenalectomy in case of cortisol-producing adrenal adenomas (CPA) or adrenocortical carcinomas (ACC). However, medical therapy is often required, e.g. to reduce perioperative risk, to control persistent hypercortisolism after surgery, or in case of advanced disease due to ectopic CS or ACC (5–7). Drugs that are typically used for this purpose are inhibitors of the adrenal steroidogenesis, glucocorticoid receptor blockers, and (in case of CD) somatostatin receptor ligands or dopamine receptor agonists (2). Among the adrenal steroidogenesis inhibitors, metyrapone and osilodrostat selectively inhibit the last enzyme of the cortisol biosynthesis, 11β-hydroxylase (CYP11B1), preventing the conversion of 11-deoxycortisol into cortisol. Metyrapone was first described in the 1950s and is still widely used today (8–11). Osilodrostat was approved by the European Medicines Agency (EMA) only recently, explaining why studies on its therapeutic efficacy are limited (6, 12–17). Furthermore, a direct comparison between metyrapone and osilodrostat has not yet been described.

The primary aim of this retrospective monocentric study was to compare the short-term efficacy of metyrapone and osilodrostat on cortisol levels in patients with endogenous CS.

## 2 Subjects and Methods

### 2.1 Subjects

**Figure 1:**
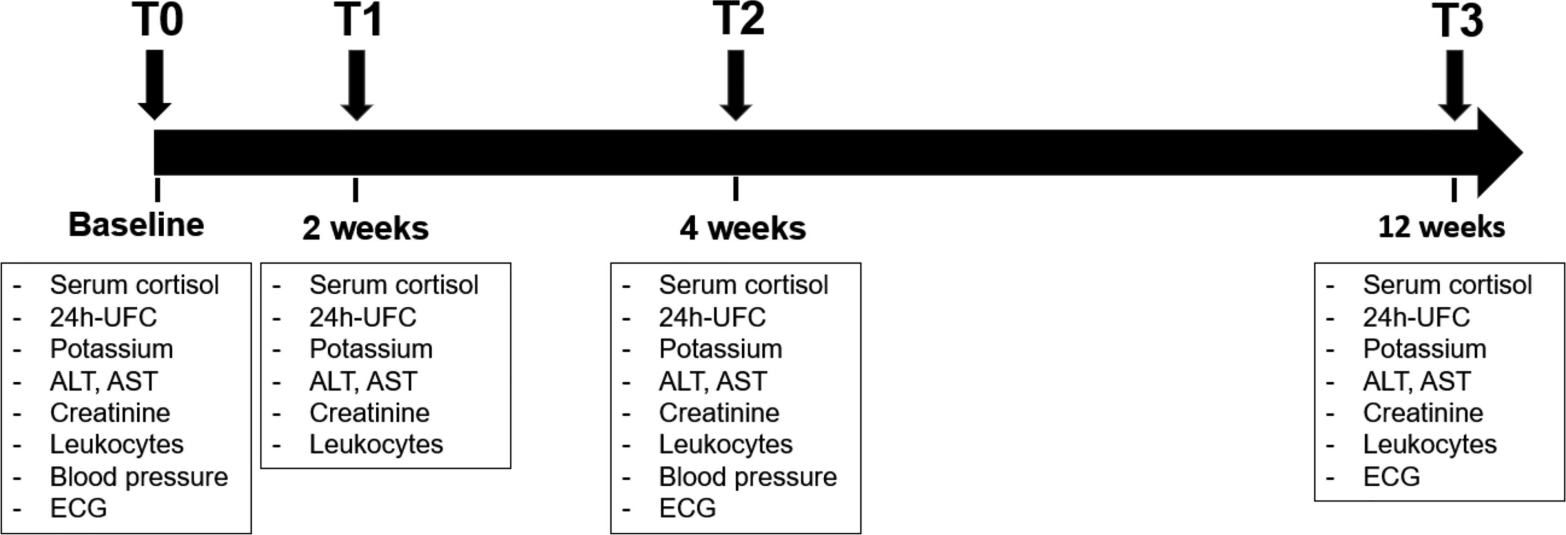
Timeline of the study with description of follow-up visit. The data analysis was performed at baseline (T0), after 2 weeks (T1), after 4 weeks (T2) and after 12 weeks (T3). ALT, alanine transaminase; AST, aspartate aminotransferase; Crea, creatinine; ECG, electrocardiography; GGT, gamma-glutamyltransferase; K, potassium; LDL, low density lipoprotein; UFC, urinary free cortisol.

Patients with endogenous CS admitted to the University Hospital Würzburg were retrospectively reviewed. Those who were treated with metyrapone or osilodrostat as monotherapy for at least four weeks between December 2017 and December 2021 were considered eligible. CS was diagnosed according to established criteria (2, 18, 19). The investigated time points are visualized in **Figure 1**. Hormonal workup with basal serum cortisol (taken from 08:00 and 10:00 a.m.), serum cortisol after an overnight 1 mg dexamethasone suppression test (DST), and 24h urinary free cortisol (UFC) was performed in all patients before any medical treatment (baseline, T0). Furthermore, biochemical routine parameters (sodium, potassium, transaminases, creatinine, cholesterol, lipoproteins, triglycerides, leukocytes), blood pressure, and electrocardiogram were also evaluated at T0. Follow-up visits were carried out after 2 weeks (T1), 4 weeks (T2), and 12 weeks (T3) of therapy. Analysis of hormonal and biochemical routine parameters (electrolytes, transaminases, creatinine, and leukocytes) was performed at T1, T2, and T3. Cholesterol, triglycerides, and blood pressure were analyzed at T2. Electrocardiography was repeated at T2 and T3 (**Figure 1**).

All patients provided written informed consent to at least one of two disease-specific clinical registries, which were approved by the local ethics committee of the University Hospital of Würzburg (approval number 88/11 for the European Network for the Study of Adrenal Tumors registry and approval number 85/12 for the Network of Excellence for Neuroendocrine Tumors registry).

### 2.2 Methods

#### 2.2.1 Hormonal Analysis

As previously performed (20, 21), commercially available analytical procedures were used for measurement of serum and salivary cortisol (the Immulite 2000 Xpi from Siemens), and for the analysis of UFC (a manual radioimmunoassay from Immuntech).

#### 2.2.2 Electrocardiogram Analysis

For the analysis of the QTc-interval the Bazzett formula [QTc=QT/√(RR/1seconds)] was used.

#### 2.2.3 Statistical Analysis

Continuous variables were reported as mean ± standard error of mean (SEM) or as median with range, whereas categorical variables were provided as numbers and percentages. Data distribution was evaluated with the Shapiro-Wilk test. Parametric and non-parametric data were analyzed with Student´s T-tests and Mann-Whitney U test, as appropriate. Dichotomic variables were analyzed with the Fisher’s exact test or the Chi-square (χ2) test. To compare the effect of metyrapone and osilodrostat on hormonal and biochemical parameters, a two-way repeated measures ANOVA was used. The delta (change) percentage from T0 to a subsequent study time point was calculated to evaluate the alteration of a parameter during the course of medical treatment with metyrapone or osilodrostat. A *p*-value < 0.05 was considered statistically significant.

Statistical Analysis was performed with SPSS version 26 (IBM Corporation, Armonk, NY, USA) and GraphPad Prism version 8 (GraphPad Software, San Diego, CA, USA).

## 3 Results

### 3.1 Study Population

In total, 7 patients with CD, 5 patients with ECS, and each 2 patients with CPA and ACC were analyzed. The metyrapone population consisted of 2 patients with CD, 4 patients with ECS, 1 patient with CPA, and 1 with ACC. The osilodrostat population included 5 patients with CD, 1 patient with ECS, 1 patient with CPA, and 1 patient with ACC (**Table 1**). Except for the ACC patients who were previously treated with a platinum-based chemotherapy (etoposide, doxorubicin, cisplatin) along with mitotane, none of these patients had previous or concomitant drug therapy for hypercortisolism. In both ACC patients mitotane was suspended at least 2 months before starting with metyrapone or osilodrostat. Previous surgery was performed in 2 patients under metyrapone (each 1 with CD and ACC) and in 3 patients under osilodrostat (2 with CD and 1 with ACC). Prior radiotherapy was performed in 1 patient with CD under metyrapone. No significant differences were observed between the 2 groups considering sex, age, basal serum morning cortisol, serum cortisol after DST, UFC, and ACTH (**Table 1**). Clinical characteristics of the entire cohort of patients at T0 are summarized in **Table 1** and reported in **Supplementary Table 1**.

**Table 1:** Clinical characteristics of the two study groups.

	Metyrapone (n=8)	Osilodrostat (n=8)	*p* value, χ^2^
Females (%)	3 (37.5%)	7 (87.5%)	0.25, χ^2^ = 1.33
Age at therapy initiation	52.1 ± 3.8	50.1 ± 4.1	0.72
**Cushing subtype** Cushing’s disease Ectopic Cushing’s syndrome Cortisol-producing adrenal adenoma Adrenocortical carcinoma	2 (25%) 4 (50%) 1 (12.5%) 1 (12.5%)	5 (62.5%) 1 (12.5%) 1 (12.5%) 1 (12.5%)	0.38, χ^2^ = 3.09
**Biochemical analysis** Basal serum cortisol (µg/dl)	27.8 ± 5.5	22.8 ± 3.5	0.45
Serum cortisol after 1-mg dexamethasone suppression test (µg/dl)	27.3 ± 8.1	12.6 ± 5.7	0.24
ACTH (ng/l)	83.9 ± 28.3	38.8 ± 12.2	0.15
Urinary free cortisol (µg/d)	758 ± 309	817 ± 644	0.93
Late-night salivary cortisol	2.3 ± 1.1	0.9 ± 0.5	0.32
Potassium (mmol/l)	3.9 ± 0.3	4.2 ± 0.2	0.53
**Blood pressure** Systolic blood pressure (mmHg)	142.0 ± 7.6	139.4 ± 4.8	0.77
Diastolic blood pressure (mmHg)	83.5 ± 4.6	83.7 ± 4.1	0.97
**Drug therapy** Therapy duration (weeks)	17.0 ± 3.4	9.5 ± 1.1	0.07
Median dose at T1 -mg (range)	1000 (500-2000)	4.0 (3.0-7.0)	–
Median dose at T2 -mg (range)	1250 (500-2000)	6.0 (4.0-20.0)	–
Median dose at T3 -mg (range)	1250 (1000-2000)	7.0 (6.0-10.0)	–

Data are reported as total number and percentage, as mean value and SEM or as median value and range. ACTH, adrenocorticotropic hormone; SEM, standard error of the mean; T1, after 2 weeks of treatment; T2, after 4 weeks of treatment; T3, after 12 weeks of treatment.

Mean time of therapy was 17.0 ± 3.4 weeks for the metyrapone group and 9.5± 1.1 weeks in the osilodrostat group (*p* < 0.0001).

Median drug dose in the metyrapone group was 1000 mg at T1 (number of patients, n = 7), 1250 mg at T2 (n = 8), and 1250 mg at T3 (n = 5). For osilodrostat, median dose was 4 mg at T1 (n = 6), 6 mg at T2 (n = 8), and 7 mg at T3 (n = 4).

### 3.2 Hormonal Values

**Figure 2:**
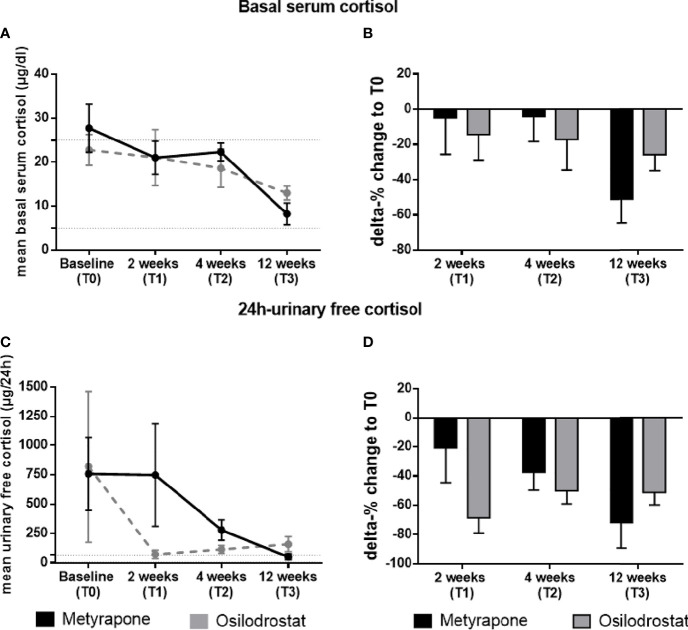
Changes of basal serum cortisol and 24h-urinary free cortisol during the follow-up in patients treated with metyrapone or osilodrostat. Changes in absolute values **(A)** and delta percentage **(B)** of morning basal serum cortisol during metyrapone or osilodrostad treatment from T0 (baseline) to 2 weeks (T1), 4 weeks (T2) and 12 weeks (T3) of therapy. Changes in absolute values **(C)** and delta percentage **(D)** of 24h-urinary free cortisol during metyrapone or osilodrostad treatment from T0 (baseline) to 2 weeks (T1), 4 weeks (T2) and 12 weeks (T3) of therapy. Absolute values are reported with mean and standard error of mean (SEM). Normal range of serum cortisol and 24h-urinary free cortisol is reported within the dotted lines in **(A, B)**.

In the metyrapone group, mean serum cortisol was 27.8 ± 5.5 µg/dL at T0 (normal range 5-25 µg/dl). During follow-up, mean serum cortisol was 21.0 ± 3.8 µg/dL at T1 (*p* = 0.61 compared with T0), 22.3 ± 2.0 µg/dL at T2 (*p* = 0.67), and 8.3 ± 2.5 µg/dL at T3 (*p* = 0.007) (**Figure 2A**). In the osilodrostat group, mean serum cortisol was 22.8 ± 3.5 µg/dL at T0, 21.1 ± 6.4 µg/dL at T1 (*p* = 0.99 compared with T0), 18.7 ± 4.3 µg/dL at T2 (*p* = 0.82) and 13.0 ± 1.6 µg/dL at T3 (*p* = 0.44) (**Figure 2A**).

Compared to T0, at T1 serum cortisol decreased by 4.9% in patients treated with metyrapone, and by 14.4% in patients treated with osilodrostat (*p = 0.63)* (**Figure 2B**). This difference was comparable to the results at T2 (-4.2% for metyrapone and -17.2% for osilodrostat, always compared to T0, *p = 0.57*). At T2, serum cortisol levels were below 25 µg/dL in 5/8 (62.5%) patients under metyrapone and in 5/8 (62.5%) patients under osilodrostat. At T3, a more pronounced decrease of cortisol was found in the metyrapone than in the osilodrostat group (-51.1% *vs* -25.8%, always compared to T0, *p = 0.23*; **Figure 2B**). At this last time point, all patients under metyrapone (5/5) and under osilodrostat (4/4) presented with a morning serum cortisol below 25 µg/dl.

Mean UFC (normal range 0-70 µg/d) in the metyrapone group was 758 ± 309 µg/d at T0. At T1, T2 and T3 was 748 ± 434 µg/d (*p* = 0.99 compared to T0), 281 ± 87 µg/d (*p* = 0.73) and 53 ± 25 µg/d (*p* = 0.62). On the other hand, mean UFC under osilodrostat at T0 was 817 ± 644 µg/d. During follow-up, UFC levels were 74 ± 36 µg/d at T1 (*p* = 0.44), 117 ± 34 µg/d at T2 (*p* = 0.41) and 131 ± 55 µg/d at T3 (*p* = 0.55) (**Figure 2C**). From T0 to T1, UFC decreased more, but not significantly, in the osilodrostat group than in the metyrapone group (-21.3% *vs* -68.4%, *p* = 0.15) (**Figure 2D**). Comparing the two groups directly from T0 to T2, both groups showed a more comparable decrease of UFC (-37.3% under metyrapone *vs* -50.1% under osilodrostat, *p* = 0.59). At this time point, 0/6 patients treated with metyrapone and 3/7 patients (42.9%) under osilodrostat had a normalized UFC. At T3, the delta change of UFC from baseline was more pronounced in the metyrapone group (-71.5% *vs* -51.5%, *p* = 0.40) (**Figure 2D**). Moreover, 2/3 patients (66.7%) under metyrapone had a normalized UFC, compared to 2/4 patients (50%) of the osilodrostat group.

In order to prevent adrenal insufficiency, a “block and replace” therapy with hydrocortisone was initiated in a subgroup of patients. In the osilodrostat group, 2/8 patients received hydrocortisone at T2, facing 3/8 patients at T3. None of the patients under metyrapone received hydrocortisone at T2, whereas 3/5 patients had hydrocortisone at T3. Of note, no difference in UFC levels was identified by including or excluding patients with block and replace therapy in the analysis.

### 3.3 Blood Pressure and Antihypertensive Drugs

**Figure 3:**
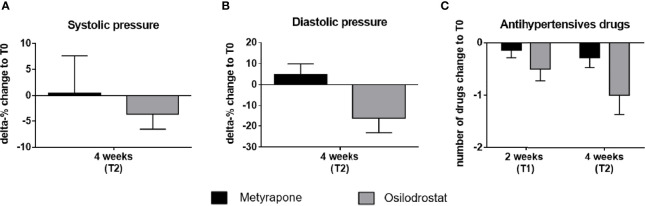
Delta percentage of systolic and diastolic blood pressure and change in number of antihypertensive drugs under metyrapone or osilodrostat treatment during follow-up compared with baseline. Changes in percentage from T0 (baseline) of **(A)** systolic, **(B)** diastolic blood pressure after 4 weeks (T2) of metyrapone or osilodrostat therapy compared with baseline (T0). **(C)** Changes in number of anti-hypertensive drugs after two weeks (T1) and 4 weeks (T2) of treatment in comparison to baseline.

A reduction of both systolic and diastolic blood pressure was observed at T2 compared to T0 in the osilodrostat group at T2 (systolic -3.7%, mean 134.2 ± 5.7 mmHg, *p* = 0.07; diastolic -16.2%, 69.2 ± 6.6 mmHg, *p* = 0.07), whereas in the metyrapone group the systolic pressure did not change relevantly and the diastolic slightly increased (systolic +0.5%, 142.5 ± 6.9 mmHg, *p* = 0.12; diastolic +4.9%, 88.7 ± 4.2 mmHg, *p* = 0.35) (**Figures 3A, B**).

The effect of osilodrostat on the blood pressure allowed a mean reduction of one antihypertensive drug at T1 (**Figure 3C**).

### 3.4 Adverse Events

Metyrapone was discontinued after 4 weeks in 2 patients (both ECS) because of adverse events (asthenia and dizziness). 1 patient under metyrapone was lost to follow-up before T3. In the osilodrostat group, the therapy was discontinued in 1 patient with CD at T2 because of adverse events (depression, asthenia, and nausea), and in 2 additional patients after tumor resection (1 CD and 1 ECS). Another patient under osilodrostat did not show up at T3 for unknown reasons and was lost to follow-up.

At T1, 1 patient under metyrapone and one under osilodrostat required potassium replacement therapy. At T2 and T3, two patients under metyrapone and 3 patients under osilodrostat required potassium replacement therapy. Analysis of potassium levels was performed only in patients without potassium replacement therapy. No significant differences in potassium levels were identified at T1 and T2. However, at T3, no substantial changes in potassium levels was identified in patients under metyrapone (-1.5% from T0, 4.3 ± 0.4 mmol/L, *p* = 0.99), while in the osilodrostat group an increase (+9.6% from T0, 4.7 ± 0.1 mmol/L, *p* = 0.43) was detected.

**Figure 4:**
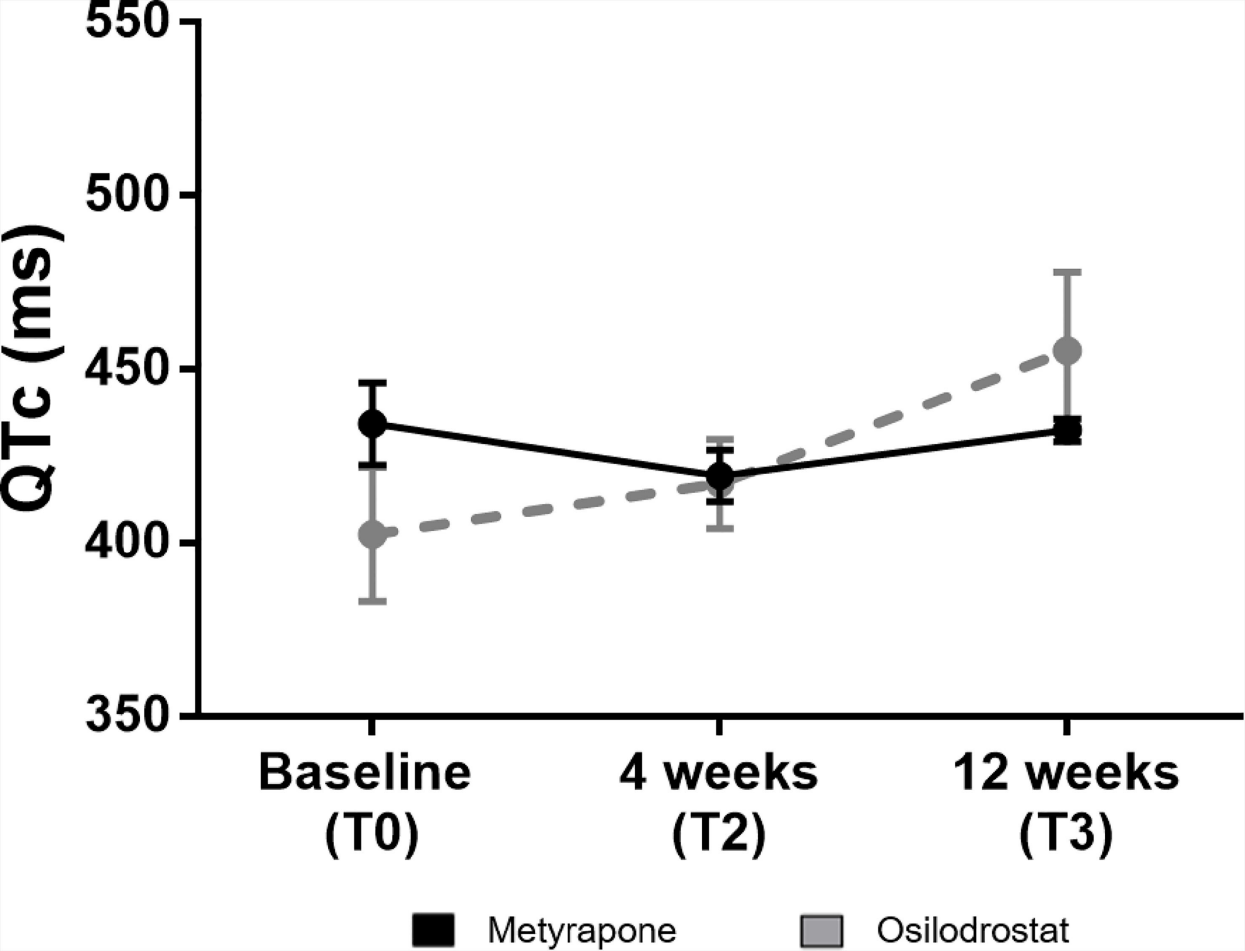
QTc-interval at baseline and during follow-up under metyrapone or osilodrostat treatment. Changes in absolute values of QTc-interval in electrocardiography under metyrapone or osilodrostat treatment. Electrocardiogram and QTc-interval analysis was performed at 4 weeks (T2) and at 12 weeks (T3). Values are reported with mean and standard error of mean (SEM).

As reported in **Figure 4**, a progressive increase of the QTc-interval was identified in the osilodrostat group, but not in the metyrapone one (455 ± 23 ms vs 432 ± 3 ms). Of note, in 1 patient under osilodrostat it was necessary to interrupt the therapy at T3 because of a QTc of 503ms.

Regarding aspartate aminotransferase (AST) and alanine aminotransferase (ALT), no substantial differences between the metyrapone and osilodrostat group were observed at T1, T2 and T3 (**Supplementary Figures 1A, B**).

We observed a not clinically relevant increase in creatinine levels in both groups (at T3 from T0, +2.4% under metyrapone, *vs* +15.3% under osilodrostat; **Supplementary Figure 1C**).

## 4 Discussion

We performed a retrospective analysis of patients with CS comparing the short-term effects of metyrapone and osilodrostat on hypercortisolism. Our data suggest that osilodrostat could reduce cortisol levels more rapidly than metyrapone, thereby allowing a better blood pressure control. Nevertheless, adverse effects like QTc prolongation under osilodrostat need to be carefully evaluated during therapy.

The efficacy of osilodrostat in different forms of CS was highlighted in previous studies, with a cortisol-normalization achieved 15 to 44 days after treatment initiation (6, 12–14, 16). In few of these cases, however, patients were previously or concomitantly treated with other drugs for hypercortisolism (13, 17). In our cohort, a reduction in UFC was obtained already after 2 weeks of therapy with osilodrostat, with a normalization of cortisol levels after 4 weeks in 42.9% of patients. This was achieved with a relative low dose of osilodrostat (6 mg/day), considering that a mean dose of 10 mg/day was reported in more than half of the patients in the phase III LINC3 trial (6). On the contrary, the metyrapone-dose was relatively high compared with a previous prospective study, in which mean doses of 750 mg and 1000 mg were reported after 1 and 3 months of treatment (8). Although the applied drug dosages of osilodrostat and metyrapone could not be directly compared, with this study we demonstrated that cortisol could be normalized with a relatively low dose of osilodrostat. Moreover, a faster decrease of UFC was achieved with osilodrostat, indicating that osilodrostat might have a superior short-time efficacy compared to metyrapone. However, this result needs to be further validated in larger (ideally prospective) studies.

The current study illustrated that metyrapone has an increasing efficacy over time. In detail, after a mild decrease of cortisol levels after 2 weeks and 4 weeks, an impressive reduction in UFC and a normalization of cortisol levels in 66.7% of the patients were identified after 12 weeks of therapy. This is in accordance with a previous report, where a 70% cortisol normalization rate was observed after 3 months (8).

During follow-up, we found a more pronounced decrease in both systolic and diastolic blood pressure under osilodrostat compared to metyrapone. A lower number of antihypertensive drugs under osilodrostat was observed after 4 weeks of treatment. In line with a previous study (8), metyrapone did not show a significant impact on blood pressure.

Hypokalemia is a well-known adverse effect of both metyrapone and osilodrostat. In fact, the inhibition of CYP11B1 indirectly causes an increase in steroid precursors with mineralocorticoid activity (6, 8). Aware of this adverse effect, we routinely performed potassium controls, and 2 patients under metyrapone and 3 patients under osilodrostat received an oral potassium replacement therapy. However, none of the patients presented severe hypokalemia (potassium <2.5 mmol/L). In patients receiving oral supplementation, potassium levels increased to the normal range.

A QTc-interval prolongation was described to be a relevant adverse event of osilodrostat, affecting 4% of the patients (6). In the present analysis, the QTc-interval increased over time as well. After 12 weeks of therapy, a mean QTc-interval of 455 ms was identified; in 1 patient, drug discontinuation was necessary due to a QTc interval of 503 ms. Accordingly, periodical controls with ECG under osilodrostat are recommended to identify relevant QTc prolongations. Of note, no significant QTc-interval prolongation was observed under metyrapone.

In the LINC 3 study, 4% of the patients showed an increase in ALT or AST under osilodrostat treatment (6). In our small study, no increase of transaminases during osilodrostat therapy was detected. This discrepancy could be due to the relatively lower dosage of osilodrostat that was used in our patients. Although hepatically metabolized, metyrapone it is not known to induce hepatic injury (2).

The current analysis has certainly relevant limitations. First, due to the rareness of CS and the very recent approval of osilodrostat by the EMA, the number of patients in both treatment groups is still very low and, therefore, the power for statistical comparisons is limited. Additionally, some patients were lost to follow-up or interrupted the therapy so at T3 only a reduced amount of patients was analyzed. Second, a retrospective design is always prone to bias and, obviously, no standardized management (e.g. regarding the dosage and follow-up visits) was implemented. This approach might have underestimated the adverse events. Third, both groups were inhomogeneous in terms of CS subtypes and clinical characteristics (although serum cortisol and UFC were comparable).

Nevertheless, to our knowledge, this is the first study that directly compared metyrapone and osilodrostat as short-term therapy of endogenous CS. The present analysis is, therefore, relevant for daily clinical practice, facilitating the choice of a certain steroidogenesis inhibitor when a prompt decrease of cortisol levels is indicated. Osilodrostat might be superior in rapidly reducing cortisol excess, but also clinical parameters like blood pressure. However, careful monitoring of the QTc intervals is required.

## Data Availability Statement

The raw data supporting the conclusions of this article will be made available by the authors, without undue reservation.

## Ethics Statement

The studies involving human participants were reviewed and approved by the University Hospital of Würzburg (approval number 88/11 for the European Network for the Study of Adrenal Tumors registry and approval number 85/12 for the Network of Excellence for Neuroendocrine Tumors registry). The patients provided their written informed consent to participate in this study.

## Author Contributions

MD and BA designed the research. MD, BA and UD performed the statistical analyses and drafted the manuscript. All authors collected samples and clinical data from patients, contributed to writing the manuscript, and approved the final version to be published.

## Funding

This work was supported by the DFG German Research Foundation Project 314061271-TRR 205 (to MF) and the European Reference Network on Rare Endocrine Conditions (Endo-ERN). This publication was supported by the Open Access Publication Fund of the University of Würzburg.

## Conflict of Interest

UD received honoraria from Recordati Rare Diseases for scientific board activities. TD received honoraria from Recordati Rare Diseases and HRA Pharma for scientific board activities.

The remaining authors declare that the research was conducted in the absence of any commercial or financial relationships that could be construed as a potential conflict of interest.

## Publisher’s Note

All claims expressed in this article are solely those of the authors and do not necessarily represent those of their affiliated organizations, or those of the publisher, the editors and the reviewers. Any product that may be evaluated in this article, or claim that may be made by its manufacturer, is not guaranteed or endorsed by the publisher.

## Supplementary Material

Supplementary Figure 1 Delta percentage of transaminase and creatinine under metyrapone or osilodrostat treatment during follow-up compared with baseline. Changes in percentage from T0 (baseline) of transaminases and creatinine throughout the study. Follow-up was performed at 2 weeks (T1), 4 weeks (T2) and 12 weeks (T3). ALT, alanine transaminase; AST, aspartate aminotransferase; GGT, gamma-glutamyltransferase.

## References

[1] Lacroix A Feelders RA Stratakis CA Nieman LK. Cushing's Syndrome. Lancet (2015) 386(9996):913–27. doi: 10.1016/S0140-6736(14)61375-1 26004339

[2] Fleseriu M Auchus R Bancos I Ben-Shlomo A Bertherat J Biermasz NR. Consensus on Diagnosis and Management of Cushing's Disease: A Guideline Update. Lancet Diabetes Endocrinol (2021) 9(12):847–75. doi: 10.1016/S2213-8587(21)00235-7 PMC8743006 34687601

[3] Chifu I Detomas M Dischinger U Kimpel O Megerle F Hahner S. Management of Patients With Glucocorticoid-Related Diseases and COVID-19. Front Endocrinol (Lausanne) (2021) 12:705214. doi: 10.3389/fendo.2021.705214 34594302 PMC8476969

[4] Pivonello R Isidori AM De Martino MC Newell-Price J Biller BM Colao A. Complications of Cushing's Syndrome: State of the Art. Lancet Diabetes Endocrinol (2016) 4(7):611–29. doi: 10.1016/S2213-8587(16)00086-3 27177728

[5] Pivonello R De Leo M Cozzolino A Colao A. The Treatment of Cushing's Disease. Endocr Rev (2015) 36(4):385–486. doi: 10.1210/er.2013-1048 26067718 PMC4523083

[6] Pivonello R Fleseriu M Newell-Price J Bertagna X Findling J Shimatsu A. Efficacy and Safety of Osilodrostat in Patients With Cushing's Disease (LINC 3): A Multicentre Phase III Study With a Double-Blind, Randomised Withdrawal Phase. Lancet Diabetes Endocrinol (2020) 8(9):748–61. doi: 10.1016/S2213-8587(20)30240-0 32730798

[7] Claps M Cerri S Grisanti S Lazzari B Ferrari V Roca E. Adding Metyrapone to Chemotherapy Plus Mitotane for Cushing's Syndrome Due to Advanced Adrenocortical Carcinoma. Endocrine (2018) 61(1):169–72. doi: 10.1007/s12020-017-1428-9 29019062

[8] Ceccato F Zilio M Barbot M Albiger N Antonelli G Plebani M. Metyrapone Treatment in Cushing's Syndrome: A Real-Life Study. Endocrine (2018) 62(3):701–11. doi: 10.1007/s12020-018-1675-4 30014438

[9] Valassi E Crespo I Gich I Rodriguez J Webb SM. A Reappraisal of the Medical Therapy With Steroidogenesis Inhibitors in Cushing's Syndrome. Clin Endocrinol (Oxf) (2012) 77(5):735–42. doi: 10.1111/j.1365-2265.2012.04424.x 22533782

[10] Verhelst JA Trainer PJ Howlett TA Perry L Rees LH Grossman AB. Short and Long-Term Responses to Metyrapone in the Medical Management of 91 Patients With Cushing's Syndrome. Clin Endocrinol (Oxf) (1991) 35(2):169–78. doi: 10.1111/j.1365-2265.1991.tb03517.x 1657460

[11] Daniel E Aylwin S Mustafa O Ball S Munir A Boelaert K. Effectiveness of Metyrapone in Treating Cushing's Syndrome: A Retrospective Multicenter Study in 195 Patients. J Clin Endocrinol Metab (2015) 100(11):4146–54. doi: 10.1210/jc.2015-2616 PMC5393433 26353009

[12] Tanaka T Satoh F Ujihara M Midorikawa S Kaneko T Takeda T. A Multicenter, Phase 2 Study to Evaluate the Efficacy and Safety of Osilodrostat, a New 11beta-Hydroxylase Inhibitor, in Japanese Patients With Endogenous Cushing's Syndrome Other Than Cushing's Disease. Endocr J (2020) 67(8):841–52. doi: 10.1507/endocrj.EJ19-0617 32378529

[13] Haissaguerre M Puerto M Nunes ML Tabarin A. Efficacy and Tolerance of Osilodrostat in Patients With Severe Cushing's Syndrome Due to non-Pituitary Cancers. Eur J Endocrinol (2020) 183(4):L7–9. doi: 10.1530/EJE-20-0557 32688343

[14] Bessiene L Bonnet F Tenenbaum F Jozwiak M Corchia A Bertherat J. Rapid Control of Severe Ectopic Cushing's Syndrome by Oral Osilodrostat Monotherapy. Eur J Endocrinol (2021) 184(5):L13–L5. doi: 10.1530/EJE-21-0147 33667191

[15] Fleseriu M Pivonello R Young J Hamrahian AH Molitch ME Shimizu C. Osilodrostat, a Potent Oral 11beta-Hydroxylase Inhibitor: 22-Week, Prospective, Phase II Study in Cushing's Disease. Pituitary (2016) 19(2):138–48. doi: 10.1007/s11102-015-0692-z PMC4799251 26542280

[16] Tabarin A Haissaguerre M Lassole H Jannin A Paepegaey AC Chabre O. Efficacy and Tolerance of Osilodrostat in Patients With Cushing's Syndrome Due to Adrenocortical Carcinomas. Eur J Endocrinol (2022) 186(2):K1–4. doi: 10.1530/EJE-21-1008 34905500

[17] Amodru V Brue T Castinetti F. Synergistic Cortisol Suppression by Ketoconazole-Osilodrostat Combination Therapy. Endocrinol Diabetes Metab Case Rep (2021) 2021:21-0071. doi: 10.1530/EDM-21-0071 34877930 PMC8686175

[18] Nieman LK Biller BM Findling JW Newell-Price J Savage MO Stewart PM. The Diagnosis of Cushing's Syndrome: An Endocrine Society Clinical Practice Guideline. J Clin Endocrinol Metab (2008) 93(5):1526–40. doi: 10.1210/jc.2008-0125 PMC2386281 18334580

[19] Fassnacht M Arlt W Bancos I Dralle H Newell-Price J Sahdev A. Management of Adrenal Incidentalomas: European Society of Endocrinology Clinical Practice Guideline in Collaboration With the European Network for the Study of Adrenal Tumors. Eur J Endocrinol (2016) 175(2):G1–34. doi: 10.1530/EJE-16-0467 27390021

[20] Detomas M Altieri B Schlotelburg W Appenzeller S Schlaffer S Coras R. Case Report: Consecutive Adrenal Cushing's Syndrome and Cushing's Disease in a Patient With Somatic CTNNB1, USP8, and NR3C1 Mutations. Front Endocrinol (Lausanne) (2021) 12:731579. doi: 10.3389/fendo.2021.731579 34489873 PMC8417750

[21] Vogg N Kurlbaum M Deutschbein T Grasl B Fassnacht M Kroiss M. Method-Specific Cortisol and Dexamethasone Thresholds Increase Clinical Specificity of the Dexamethasone Suppression Test for Cushing Syndrome. Clin Chem (2021) 67:998–1007. doi: 10.1093/clinchem/hvab056 33997885

