# Supplementary figures and images for: Metyrapone Versus Osilodrostat in the Short-Term Therapy of Endogenous Cushing’s Syndrome: Results From a Single Center Cohort Study
Source: Front Endocrinol (Lausanne). 2022 Jun 13;13:903545. doi: 10.3389/fendo.2022.903545 (PMC9235400; doi:10.3389/fendo.2022.903545)

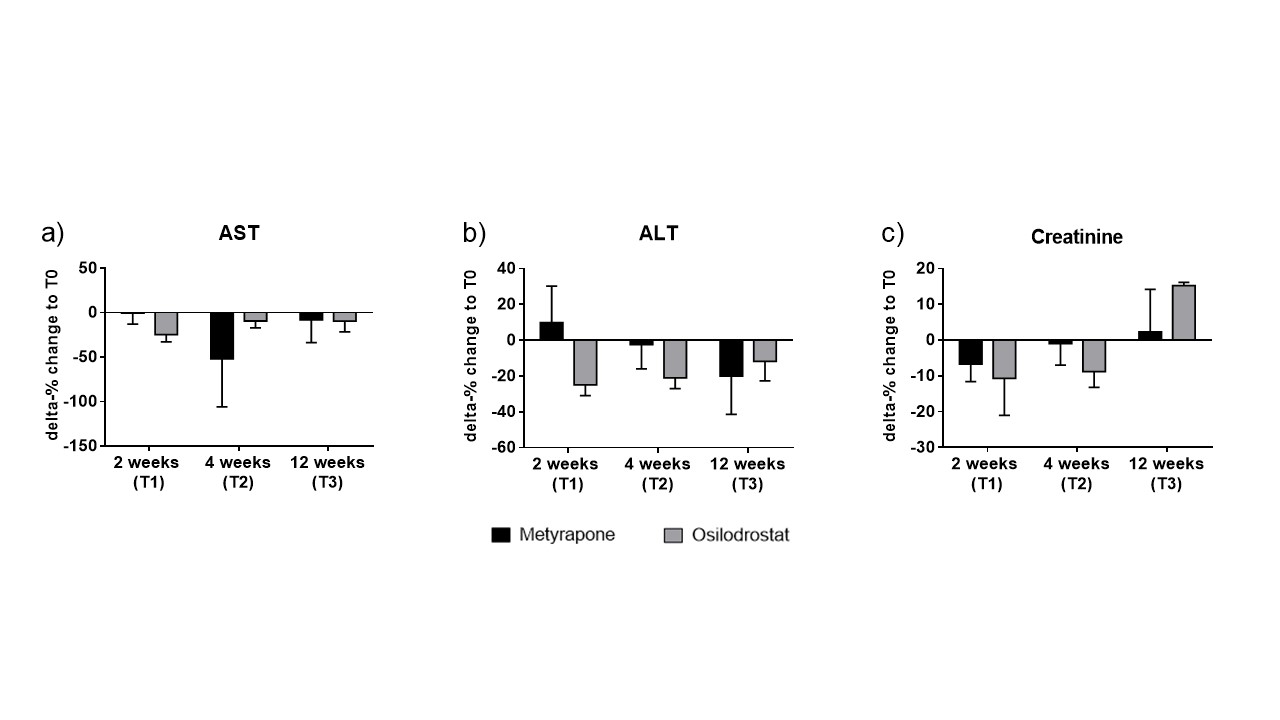

Supplement: Supplementary Figure 1 — Delta percentage of transaminase and creatinine under metyrapone or osilodrostat treatment during follow-up compared with baseline. Changes in percentage from T0 (baseline) of transaminases and creatinine throughout the study. Follow-up was performed at 2 weeks (T1), 4 weeks (T2) and 12 weeks (T3). ALT, alanine transaminase; AST, aspartate aminotransferase; GGT, gamma-glutamyltransferase. [file Image_1.jpg]
