# Supplementary material for: Metyrapone Versus Osilodrostat in the Short-Term Therapy of Endogenous Cushing’s Syndrome: Results From a Single Center Cohort Study
Source: Front Endocrinol (Lausanne). 2022 Jun 13;13:903545. doi: 10.3389/fendo.2022.903545 (PMC9235400; doi:10.3389/fendo.2022.903545)
Supplement: Supplementary file 2 [file Table_1.docx]

**Supplementary Table 1. Baseline characteristics of the entire cohort of patients**

| **Treatment** | **Patient** | **Sex** | **Age (years)** | **Year of drug therapy initiation** | **BMI**  **(kg/m^2^)** | **CS subtype** | **24h-UFC**  **(µg/d)** | **Basal serum cortisol**  **(µg/dl)** | **Potassium**  **(mmol/l)** | **Potassium**  **Substitution** | **Surgery** | **Radio-therapy** | **Chemotherapy, med. therapy** | **Striae rubrae** | **Asthenia** | **Hirsuitism**  **(Ferriman-Gallewey Score)** | **Systolic BP**  **(mmHg)** | **Diastolic BP**  **(mmHg)** | **Nr. of AH** |
| --- | --- | --- | --- | --- | --- | --- | --- | --- | --- | --- | --- | --- | --- | --- | --- | --- | --- | --- | --- |
| **Metyrapone** | Met1 | F | 39 | 2019 | 29.5 | CD | 750 | 16.5 | 4.5 | No | yes | yes | no | yes | yes | 2 | 135 | 85 | 0 |
|  | Met2 | F | 66 | 2018 | 31.2 | CD | 71 | 13.1 | 4.4 | No | no | no | no | no | yes | 1 | 145 | 75 | 3 |
|  | Met3 | F | 48 | 2017 | 26.4 | ECS | 264 | 22.8 | 4.7 | No | no | no | no | yes | yes | 5 | 107 | 72 | 1 |
|  | Met4 | M | 51 | 2017 | 24.3 | ECS | 1752 | 42.1 | 2.8 | Yes | no | no | no | yes | yes | - | 145 | 85 | 3 |
|  | Met5 | M | 51 | 2019 | 29.1 | ECS | 2342 | 56.7 | 2.8 | No | no | no | no | no | yes | - | 150 | 80 | 3 |
|  | Met6 | M | 65 | 2017 | 25.2 | ECS | 545 | 19.5 | 4.0 | No | no | no | no | yes | yes | - | 159 | 91 | 3 |
|  | Met7 | F | 45 | 2019 | 33.4 | CPA | 78 | 16.0 | 4.5 | No | no | no | no | yes | yes | 2 | 120 | 70 | 2 |
|  | Met8 | F | 55 | 2020 | 25.6 | ACC | 170 | 35.3 | 3.9 | No | yes | no | EDP + Mitotane | no | no | 1 | 175 | 110 | 4 |
| **Osilodrostat** | Osi1 | M | 32 | 2020 | 27.4 | CD | 240 | 15.6 | 4.0 | No | no | no | no | yes | yes | - | 115 | 70 | 0 |
|  | Osi2 | F | 41 | 2021 | 41.0 | CD | 91 | 17.2 | 4.9 | No | no | no | no | no | yes | 2 | 140 | 82 | 1 |
|  | Osi3 | F | 50 | 2020 | 38.1 | CD | 74 | 18.4 | 4.4 | No | yes | no | no | no | yes | 3 | 150 | 103 | 3 |
|  | Osi4 | F | 51 | 2020 | 31.6 | CD | 281 | 19.0 | 4.5 | No | no | no | no | yes | yes | n.a. | 145 | 95 | 4 |
|  | Osi5 | F | 60 | 2021 | 19.2 | CD | 72 | 20.6 | 3.2 | No | yes | no | no | no | yes | 1 | 135 | 80 | 3 |
|  | Osi6 | F | 68 | 2021 | 35.9 | ECS | 5311 | 46.3 | 4.0 | Yes | no | no | no | yes | yes | 5 | 140 | 90 | 5 |
|  | Osi7 | F | 56 | 2021 | 44.6 | CPA | 71 | 20.8 | 4.5 | No | no | no | no | no | yes | 0 | 130 | 70 | 0 |
|  | Osi8 | F | 43 | 2021 | 22.3 | ACC | 400 | 24.5 | 3.8 | No | yes | no | EDP + Mitotane | yes | yes | 4 | 160 | 80 | 2 |

Abbreviations: ACC: adrenocortical carcinoma; AH: antihypertensive; BMI: body mass index, BP: blood pressure; CS: Cushing’s syndrome; CD: Cushing’s disease; CPA: cortisol producing adrenal adenoma; ECS: ectopic Cushing’s syndrome; EDP: etoposide, doxorubicin, cisplatin; F, female; M, male; n.a.: not available; Med. therapy: previous medical therapy against hypercortisolism or ACC; Radioth: radiotherapy; UFC: urinary free cortisol.
